# Genome sequence and virulence variation-related transcriptome profiles of *Curvularia lunata*, an important maize pathogenic fungus

**DOI:** 10.1186/1471-2164-15-627

**Published:** 2014-07-24

**Authors:** Shigang Gao, Yaqian Li, Jinxin Gao, Yujuan Suo, Kehe Fu, Yingying Li, Jie Chen

**Affiliations:** School of Agriculture and Biology, Shanghai Jiao Tong University, 800 Dongchuan Road, Shanghai, 200240 P. R. China; State Key Laboratory of Microbial Metabolism, Shanghai Jiao Tong University, 800 Dongchuan Road, Shanghai, 200240 P. R. China; Ministry of Agriculture Key Laboratory of Urban Agriculture (South), Shanghai Jiao Tong University, 800 Dongchuan Road, Shanghai, 200240 P. R. China

**Keywords:** *Curvularia lunata*, Genome sequencing, RNA-seq, Pathogenicity variation

## Abstract

**Background:**

*Curvularia lunata* is an important maize foliar fungal pathogen that distributes widely in maize growing area in China. Genome sequencing of the pathogen will provide important information for globally understanding its virulence mechanism.

**Results:**

We report the genome sequences of a highly virulent *C. lunata* strain. Phylogenomic analysis indicates that *C. lunata* was evolved from *Bipolaris maydis* (*Cochliobolus heterostrophus*)*.* The highly virulent strain has a high potential to evolve into other pathogenic stains based on analyses on transposases and repeat-induced point mutations. *C. lunata* has a smaller proportion of secreted proteins as well as *B. maydis* than entomopathogenic fungi. *C. lunata* and *B. maydis* have a similar proportion of protein-encoding genes highly homologous to experimentally proven pathogenic genes from pathogen-host interaction database. However, relative to *B. maydis*, *C. lunata* possesses not only many expanded protein families including MFS transporters, G-protein coupled receptors, protein kinases and proteases for transport, signal transduction or degradation, but also many contracted families including cytochrome P450, lipases, glycoside hydrolases and polyketide synthases for detoxification, hydrolysis or secondary metabolites biosynthesis, which are expected to be crucial for the fungal survival in varied stress environments. Comparative transcriptome analysis between a lowly virulent *C. lunata* strain and its virulence-increased variant induced by resistant host selection reveals that the virulence increase of the pathogen is related to pathways of toxin and melanin biosynthesis in stress environments, and that the two pathways probably have some overlaps.

**Conclusions:**

The data will facilitate a full revelation of pathogenic mechanism and a better understanding of virulence differentiation of *C. lunata*.

**Electronic supplementary material:**

The online version of this article (doi:10.1186/1471-2164-15-627) contains supplementary material, which is available to authorized users.

## Background

The maize leaf spot caused by *C. lunata* have ever made tremendous yield loss of maize in 11 provinces of maize growing areas in China since the 1990s. Especially in northern China, for instance, it occurred over 192000 hm^2^ and led to 8 million kg yield loss in Liaoning province in 1996 [1–3]. Many research projects have been designed to discover the disease occurrence pattern, resistance breeding and integrated control of the disease. As the application of resistance varieties containing tropic and sub-tropic germplasms in large growing areas, the incidence of disease infection and its severity were declined massively and less damage was observed in field. However, in recent years, the disease has bounced back again and caused serious damages in some maize growing areas such as Liaoning, Anhui and Henan province etc. The main cause of the disease recurrent was expected to link to large area of monoculture with same or similar resistant germplasm which then become a matrix to induce pathogen virulence variation.

*C. lunata* is a major organism causing the leaf spot disease in maize [4], but *Cochliobolus lunatus* as teleomorph of *C. lunata* is expected to form when the pathogen suffer stress condition in maize field in most cases. The teleomorph is not a major form to cause the foliar disease. *C. lunata* has 5 pathogenic types from high to low virulence in China. The distribution of pathogenic types varies in different maize growing areas. *C. lunata* has broad host range including maize, wheat, barley and sorghum and other grasses. Meanwhile a multiple virulence factors have been demonstrated to be involved in pathogen infection to maize, such as cellulose [5], non-host specific toxin (methyl 5-(hydroxymethyl)-furan-2-carboxylate) [6], melanin [7]. It is worth mentioning that some of virulence related genes have been successfully cloned in previous work such as *clt-1* regulating non-host specific toxin production, *brn1* being required for DHN melanin synthesis [8], two mitogen-activated protein kinases (MAPK) encoding genes (*clk1* and *clm1*) [9, 10]. Previous study preliminarily showed that *brn1* was not only involved in melanin synthesis, but also associated with toxin biosynthesis. Whereas, the role of these genes in collaborated regulation of toxins and melanin production remains unknown. In addition, several novel virulence-associated genes and large suites of enzymes such as polyketide synthases (PKS) being involved in secondary metabolism were also associated with pathogenicity in most fungal pathogens [11, 12]. However, there is no detailed knowledge to reveal the genetically synergistic regulation mechanism of those virulence-associated genes in the *C. lunata* so far.

*C. heterostrophus* (agamotype: *B. maydis*) causing a widely distributed maize fungal disease, southern leaf blight, belongs to *Cochliobolus* as well as *C. lunatus* (agamotype: *C. lunata*). As the genome of *B. maydis* has been accomplished in recent years, it provides a great deal of bioinformation for globally understanding the detailed infection mechanism of *C. lunata* and its interaction mechanism with maize. Based on previous work, it was found that *C. lunata* showed high homology with *B. maydis* in genetic evolution, thus *C. lunata* could be profiled in genome wide through comparative genome analysis. However, until now, very little information is known about developmental and pathogenic process of *C. lunata* from a global view, even though a normalized full-length cDNA library of *C. lunata* was constructed in the previous study [13]. As we mentioned, the production of melanin and non-host specific toxin may share the common gene regulation network or overlap some node genes responsible for cross-talking between both regulation network systems. Three velvet-like protein-encoding genes being interactive with *brn1* gene were successfully screened in *C. lunata* by yeast two hybrid [14]. Further work showed that velvet-like protein was involved in the regulation of pathogen pathogenicity [15].

PAMP (pathogen associated molecular pattern) model is widely applied in a group of crops to illustrate basic immunity or mechanism of *C. lunata*-maize interaction. Previous study showed that planting resistant maize varieties for long term in a certain areas would induce virulence variation of *C. lunata* and some genes served as hallmarks displaying the virulence variation, most of which are secreted protein-encoding genes. Similarly a large amount of secreted proteins have been identified in *B. maydis* (*C. heterostrophus*), which are responsible for specific interaction of *B. maydis* with maize germplasm [16]. Taken together, it is expected that multiple virulence-associated genes control pathogen infection in host plants. Although recently the functions of virulence-associated genes were characterized, the details on the relations among the expression modes of these genes still remain unclear, owing to lack of global understanding of *C. lunata* genome sequence. However, the global analysis on genome sequence of the pathogen *C. lunata* will be imperative and facilitate more rapid identification of genes responsible for pathogenicity and pathogen-plant interactions. Hence, in this work, firstly the draft genome sequence of *C. lunata* CX-3 with high virulence was presented, and comparative analyses of genome repertoire among *C. lunata* CX-3 isolated from maize, *C. lunata* m118 isolated from sorghum and *B. maydis* C5 were conducted in pathogen proliferation and development, virulence and genetic variation, secondary metabolism, plant-pathogen interactions, signaling pathway and detoxification. Secondly, RNA-seq was applied to comparatively analyze transcriptional profiles of *C. lunata* WS18 with weak virulence and its virulence-increased variant WS18-Pob21-11. This work will provide abundant information for globally uncovering the infection mechanism of *C. lunata* to maize, thereby the new insight to the pathogen development and infection mechanism would be generated in genome wide. Last but not least comparative genomic and transcriptome analysis for virulence variation of *C. lunata* will contribute to better understanding what is required to develop flexible strategies to control the pathogen infection.

## Results

### Genome sequencing and general features

The genome of *C. lunata* CX-3 was sequenced (65× coverage) with Illumina sequence technology; the sequenced reads were assembled into 340 scaffolds (N50, 788 kb) with a total size of 35.5 Mb, similar to *B. maydis* C5 (36.5 Mb) (GenBank: AIDY00000000), but bigger than *C. lunata* m118 (31.2 Mb) (JGI: 403758) (Table 1). By mapping 23390 unigenes to the scaffolds of *C. lunata* CX-3, the completeness of *C. lunata* CX-3 genome was estimated to be > 99%. By prediction, *C. lunata* CX-3 genome encoded a total of 11234 protein genes similar to *C. lunata* m118 and fewer than *B. maydis* C5 (Table 1). Similarly, the proportion of genes encoding secreted proteins in *C. lunata* CX-3 was 7.5% (840 proteins), similar to 7.6% in *C. lunata* m118 (834), but higher than 6.7% in *B. maydis* C5 (886) (Table 1). Although the proportions of secreted proteins in the two plant pathogens were close to 7-10% in other sequenced plant pathogens such as *Magnaporthe oryzae* [17, 18], they were far lower than that in three insect pathogenic fungi (16.2% in *Cordyceps militaris*, 17.6% in *Metarhizium anisopliae* and 15.1% in *Metarhizium acridum*) [19, 20].

**Table 1:** **Comparison of genome features between** ***C. lunata*** **and** ***B. maydis***

Features	***C. lunata***CX-3	***C. lunata***m118	***B. maydis***C5
Assembly size (Mb)	35.5	31.2	36.5
Scaffolds	340	171	89
GC (%)	50.22	50.9	50.0
Repeated sequences (%)	2.44	1.07	1.33
Protein-coding genes	11,234	11,004	13,316
Gene density (genes per Mb)	314.7	353.8	280
Mean gene length (Bp)	1,448	1,429	1,284
Secreted proteins	840	834	886

*B. maydis* = *C. heterostrophus*.

**Figure 1:**
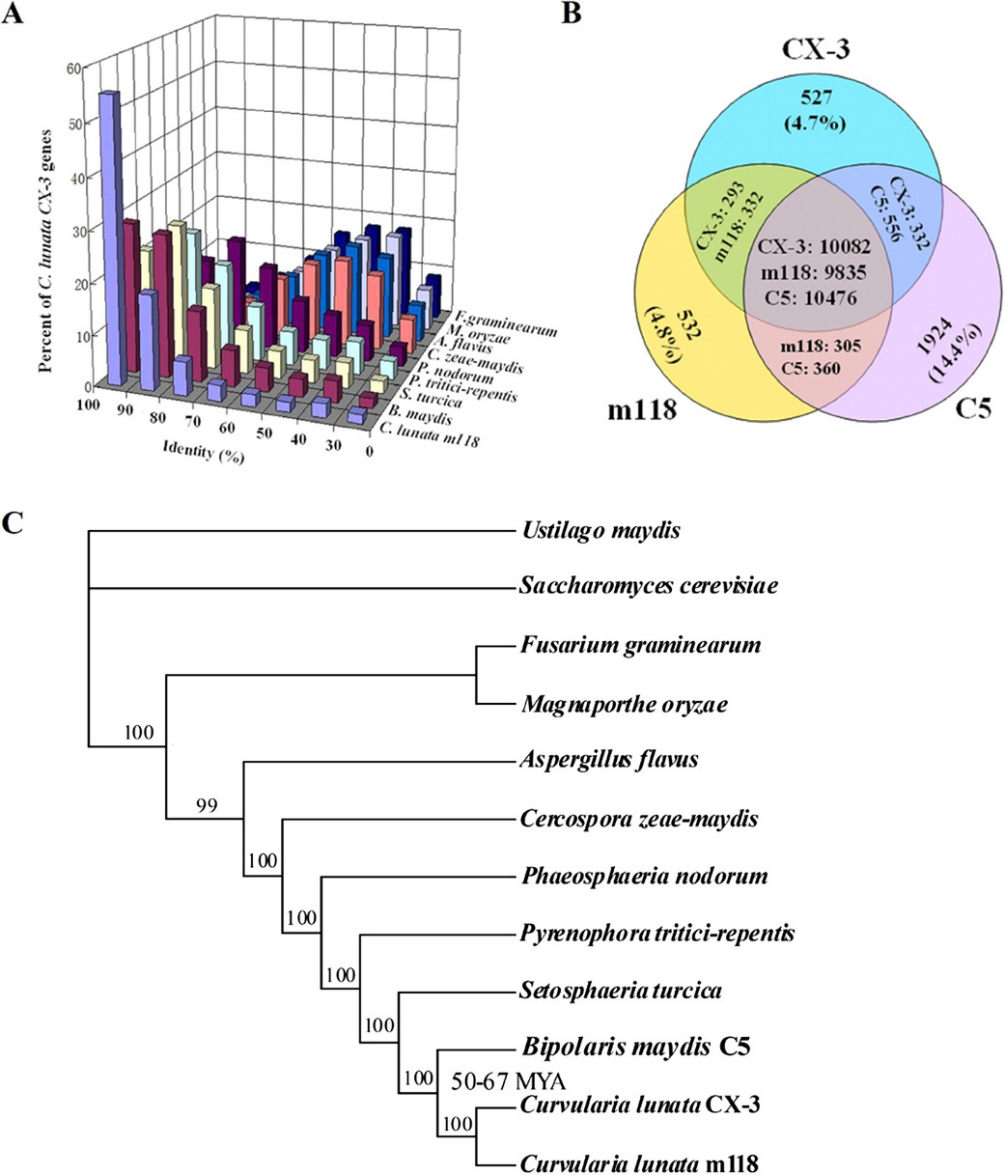
**Comparative genomics and evolutionary analysis of** ***C. lunata.*** **(A)** Amino acid sequence identity of *C. lunata* CX-3 with other fungi. **(B)** Reciprocal Blast analysis of the protein sequences among the three pathogenic fungi *C. lunata* CX-3, *C. lunata* m118 and *B. maydis* C5 with a cut-off *E* value of 1e-5. CX-3, *C. lunata* CX-3; m118, *C. lunata* m118; C5, *B. maydis* C5. In relative to *B. maydis* C5, *C. lunata* CX-3 and *C. lunata* m118 have 820 (7.3%) and 864 (7.9%) species-specific genes; In relative to *C. lunata*, *B. maydis* C5 has 1924 (14.4%) species-specific genes. *C. lunata* CX-3 and *C. lunata* m118 have 859 and 837 strains-specific genes, respectively. **(C)** A phylogenetic tree constructed with the Dayhoff amino acid substitution model representing the evolutionary relationships of *C. lunata* and other fungi. MYA, million years ago. *B. maydis* = *C. heterostrophus*.

Approximately 55% of *C. lunata* CX-3 genes have >90% of amino acid sequence identity with *C. lunata* m118 (Figure 1A). Although the sexual stages of both *C. lunata* and *B. maydis* are *Cochliobolus* species, only about 30% of *C. lunata* CX-3 genes have >90% of amino acid sequence identity with *B. maydis* C5, and the proportion is far lower that between *M. anisopliae* and *M. acridum* (more than 50%) [19]. Comparative genomic analysis showed that *C. lunata* CX-3, *C. lunata* m118 and *B. maydis* C5 have a large number of species-specific and strains-specific genes (Figure 1B). 10082, 9835 and 10476 orthologous core genes and 820, 864 and 1924 species-specific genes were identified in *C. lunata* CX-3, *C. lunata* m118 and *B. maydis* C5, respectively. The proportion of species-specific genes in *C. lunata* CX-3 (about 7.3%) was similar to *C. lunata* m118 (7.9%), but far lower than *B. maydis* C5 (14.4%) (Figure 1B), showing that *C. lunata* CX-3 has more differences with *B. maydis* C5 followed by *C. lunata* m118. In addition, 859 and 837 strains-specific genes were also identified in *C. lunata* CX-3 and *C. lunata* m118, respectively (Figure 1B), showing that two different *C. lunata* strains have distinct difference in genome. Genomic islands (GIs) are part of a genome and contain at least three contiguous gene-encoding proteins that do not exist in the reference genome [21]. GIs have many functions, especially they are involved in symbiosis or pathogenesis and may help organism’s adaptation. GIs are classed into many sub-classes based on their function. For example, GIs associated with pathogenesis are often called as pathogenicity islands (PAIs), and GIs containing many antibiotic resistant genes are referred to as antibiotic resistance islands (ARIs). It was found by reciprocal analysis that *C. lunata* CX-3 has 40 and 16 GIs separately in comparison to *C. lunata* m118 and *B. maydis* C5, which probably play key roles in virulence.

Identity analysis between orthologous proteins shows that *C. lunata* CX-3 has an average about 85.9% amino acid identity with *C. lunata* m118, higher than with other plant pathogenic fungi such as *B. maydis* C5 (78.6%), *Setosphaeria turcica* (JGI: 401988) (75.8%) and *Pyrenophora tritici-repentis* (GenBank: AAXI00000000) (70.9%), especially far higher than with *M. oryzae* (GenBank: AACU00000000) orthologs (47.9%) (Table 2). Therefore, *C. lunata* and *B. maydis* are more close than three *Aspergillus* species (*A. oryzae*, *A. nidulans* and *A. fumigatus*) with an average of 68% protein sequence identity [21], but more diverged than two *Metarhizium* spp. (*M. anisopliae* and *M. acridum*) with an average of 89.8% protein sequence identity [20]. A phylogenomic analysis revealed that *C. lunata* CX-3 and *C. lunata* m118 diverged 24.3–50.4 million years ago (MYA) and diverged from their most closely related maize pathogen *B. maydis* 50.4–67.0 MYA (Figure 1C). In additionally, *B. maydis* diverged from *S. turcica* 67.1-93.0 MYA, which diverged from *P. tritici-repentis* 93–136 MYA. Therefore, the lineage leading to *C. lunata* (*C. lunatus*) diverged from *B. maydis* (*C. heterostrophus*) around Cretaceous Extinction (65 MYA), and the *Cochliobolus* lineage diverged from other plant pathogenic fungi after the Triassic-Jurassic Event (200 MYA) [22]. It was also showed in Figure 1C that *M. oryzae* and *Fusarium graminearum* diverged 274–403 MYA (before the Triassic-Jurassic Event), which was much earlier than the divergence time between *Cochliobolus* lineage and other plant pathogenic fungi.

**Table 2:** **Genome-wide analysis of** ***C. lunata*** **CX-3 gene sets**

Characteristics	CX-3	CCC core ^a^	CX-3-m118 specific ^b^	CX-3-C5 specific ^c^	CX-3 specific
Protein-encoding genes	11234	10082	293	332	527
Protein families	2830	2780	71	66	48
Secreted proteins	840	736	41	23	40
PHI genes^d^	1904	1880	2	10	12
Transposases	161	78	5	34	44
Proteases	516	502	5	4	5
Glycoside hydrolases	219	216	0	1	2
MFS transporters	252	252	0	0	0
ABC transporters	47	47	0	0	0
P450s	147	147	0	0	0
GPCRs	129	128	1	0	0
Pth11-like GPCRs	37	0	0	0	0
Protein kinases	153	153	0	0	0
Fungal specific transcription factors	222	209	9	2	2
Backbone genes for secondary metabolism	34	34	0	0	0
Orthologs in *C. lunata* m118	10375	10082	293	NA	NA
Orthologs in *B. maydis* C5	10414	10082	NA	332	NA
Orthologs in *S. turcica*	10268	9833	125	238	72
Orthologs in *P. tritici-repentis*	9979	9656	111	144	68
Orthologs in *M. oryzae*	8462	8320	56	62	24
Identity to *C. lunata* m118 orthologs	85.9%	86.4%	68.8%	NA	NA
Identity to *B. maydis* C5 orthologs	78.6%	79.1%	NA	64.2%	NA
Identity to *S. turcica* orthologs	75.8%	76.5%	53.3%	62.7%	57.2%
Identity to *P. tritici-repentis* orthologs	70.9%	71.6%	50.4%	52.8%	47.0%
Identity to *M. oryzae* orthologs	47.9%	48.0%	38.4%	39.3%	36.9%

CX-3: *C. lunata* CX-3. ^a^CCC core: *C. lunata* CX-3, *C. lunata* m118 and *B. maydis* C5 genes grouped with a cutoff *E* value of 1e-5 during reciprocal Blast analysis. ^b^CX-3-m118 specific: *C. lunata* CX-3 and *C. lunata* m118 specific genes in relative to *B. maydis* C5 grouped with a cutoff *E* value of 1e-5. ^c^CX-3-C5 specific: *C. lunata* CX-3 and *B. maydis* C5 specific genes in relative to *C. lunata* m118 grouped with a cutoff *E* value of 1e-5. ^d^PHI genes, pathogen-host interaction genes identified by Blast analysis against the PHI database with a cutoff *E* value of 1e-5. Identity was estimated using amino acid sequences. GPCR, G-protein-coupled receptor; MFS, major facilitator superfamily; NA, not available. *B. maydis* = *C. heterostrophus*.

Pfam matches were performed to identify 2830 conserved protein families containing 8471 proteins in *C. lunata* CX-3 genome close to *C. lunata* m118 with 2827 families containing 8181 proteins and slightly lower than *B. maydis* C5 with 2860 families containing 8886 proteins. In phytopathogenic fungi, family expansions were identified in glycoside hydrolases (GHs), cutinases and pectin lyases compare to insect pathogenic fungi [19], showing the important roles of GHs, cutinases and pectin lyases in pathogenicity of phytopathogenic fungi. As a plant pathogen, *C. lunata* CX-3 has a large number of GHs, cutinases and pectin lyases (Additional file 1: Table S1). In comparison with *C. lunata* m118, *C. lunata* CX-3 has family expansions in transposase, fungal specific transcription factors, major facilitator superfamily, ABC superfamily, cytochrome P450, protein kinase, serine protease, subtilisin and glucosidase, and family constrictions in G-protein coupled receptor and heterokaryon incompatibility (Additional file 1: Table S1 and S2), which are expected to be crucial for the fungal survival in varied stress environments.

To excavate potential pathogenic genes, a Blast search of the *C. lunata* CX-3 genome was performed against the pathogen-host interaction (PHI) database, which collected the experimentally proven genes affecting the outcome of pathogen-host interactions of fungi, bacteria and oomycetes [23]. 17.0% of predicted genes in *C. lunata* CX-3 (1904), identified by a Blast search of *C. lunata* CX-3 genome against the PHI database, are putatively related to pathogen-host interaction. The percentage of PHI genes in *C. lunata* CX-3 (17.0%) is close to other plant pathogenic fungi (16.6% in *C. lunata* m118, 15.0% in *B. maydis* C5, 17.0% in *F. graminearum* and 17.7% in *Aspergillus flavus*) (Additional file 1: Table S2).

### Transposases and repeat-induced point mutation

Like rice pathogen *Magnaporthe grisea*, *C. lunata* has a high degree of genetic variability and prone to form novel pathogenic variants to infect the resistant host. Transposases and repetitive elements make a greater contribution to genetic instability and pathogenic variation [24]. The transposable elements are closely associated with the virulence of most ascomycetes including *C. lunata*. Thus, an understanding of the change of transposons and repeat elements in *C. lunata* not only provides an insight into their impact on genome evolution and also contributes to shedding light on mechanisms of pathogenic variation.

The number of transposases in *C. lunata* CX-3 (161) was far more than *C. lunata* m118 (24) and *B. maydis* C5 (97) (Additional file 1: Table S3)*.* Out of 161 transposases in *C. lunata* CX-3, 83 were DNA transposases, 54 LTR transposases and 24 LINE transposases. However, *C. lunata* m118 genome lacks LINE transposases. Most of these transposases in *C. lunata* CX-3 are rich in DNA (79), LINE (24) and LTR (54) transposases, of which subclasses such as TcMar (70), Tad1 (22), *Copia* (18) and Gypsy (28) are much more abundant than other subclasses (Additional file 1: Table S3). Transcriptome analysis also showed that 3 transposase genes (CL00030, CL10102 and CL00391) were up-regulated in *C. lunata* with higher virulence.

**Figure 2:**
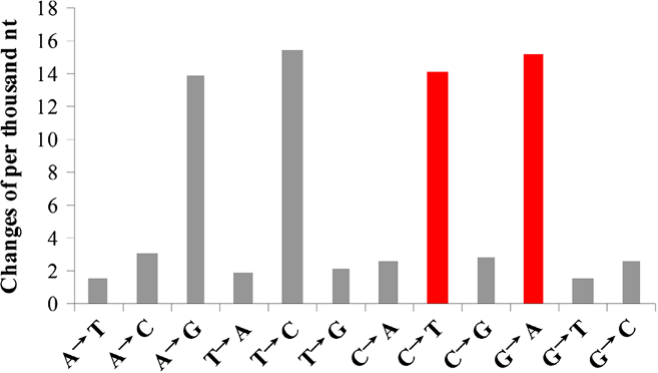
**Nucleotide mutation rates in** ***C. lunata*** **CX-3 paralogous genes showing >80% identity in paired protein sequences.** For calculations, the gene of each pair with >90% nucleotide sequence similarities to the ortholog from *S. turcica* was used as the reference.

Repeat-induced point mutation (RIP) in ascomycete fungi is a genome defense, which hyper-mutates repetitive DNA and limit the accumulation of transposable elements [25]. RIP preferentially introduces CA/G dinucleoutides to TA during the sexual cycle in many fungi, such as *Metarhizium* spp. and *Trichoderma* spp. etc. 68 paired *C. lunata* CX-3 paralogous genes showing >80% nucleotide sequence identities were used to estimate nucleotide mutation frequencies. The results showed that there was strong mutation bias: C:G to T:A (Figure 2), which is introduced by repeat induced point mutations (RIP) [26]. The *C. lunata* CX-3 genome contains many transposable elements, suggesting that they can escape damage by RIP.

### Protein families involved in degrading plant cell wall and cuticle

*C. lunata* as a plant pathogen need to break through the passive defense (cell wall, cuticle) of plant for successful infection [27]. Therefore, it would be expected to produce and secrete large numbers of extracellular degrading enzymes to degrade the plant cell wall and cuticle such as glycoside hydrolases, pectinase and cutinase, in which glycoside hydrolase (GH) is one of the representative. The number of GH genes in *C. lunata* CX-3 (219) is close to *C. lunata* m118 (214) and the average in plant pathogenic fungi (211) and less than *B. maydis* C5 (241) (Additional file 1: Table S4), but far larger than that in three insect pathogenic fungi (159 in *M. anisopliae*, 130 in *M. acridum*, 135 in *C. militaris*) [19, 20]. There is no distinct difference in the number of GH genes for each family except for GH18 and GH43 family among *C. lunata* CX-3, *C. lunata* m118 and *B. maydis* C5. 31.9% of GH genes in *C. lunata* CX-3 (75) are identified to be putative PHI genes, 34.1% in *C. lunata* m118 (83) and 34.0% in *B. maydis* C5 (82), which are close to the average in plant pathogenic fungi (31.5%) but higher than that in insect fungi *Metarhizium* spp. (about 20%) [20].

Plant pathogenic fungi contain almost all GH families of insect pathogenic fungi (*Metarhizium* spp. and *C. militaris*), but some GH families existing in *C. lunata* and other plant pathogenic fungi are not present in insect fungi, including GH6, GH7, GH45 and GH61 cellulases, GH10 xylanases, GH13 amylases, GH30 glucosylceramidases, GH62 and GH78 rhamnosidases, GH79 and GH115 glucuronidases, GH125 mannosidases [19]. The GH families of cellulases are well presented in *C. lunata* CX-3 (55), *C. lunata* m118 (53) and *B. maydis* C5 (56), including GH3, GH6, GH7, GH45 and GH61 cellulases. Among the GH families the GH3 and GH61 cellulases are the majority, but lack of GH5 cellulases. GH18 chitinases are involved in degrading insect chitin, so predictably GH18 genes are less in *C. lunata* and other plant pathogenic fungi (11 in *C. lunata* CX-3, 14 in *C. lunata* m118, 9 to 19 in other phytopathogenic fungi) than in insect pathogenic fungi (30 in *M. anisopliae* and 21 in *M. acridum*) [19]. Additionally, GH16 family of xyloglucosyl transferases for xyloglucan catabolism play an important role in digesting plant cell walls, and they are well presented in plant pathogenic fungi including *C. lunata* CX-3 (19), *C. lunata* m118 (19) and *B. maydis* C5 (21).

### Protein families for transportation

In fungal transporters, ATP-binding cassette (ABC) transporters and the major facilitator superfamily (MFS) are the two biggest superfamilies. The ABC transporters are multi-component primary active transporters, which transport both small molecules and macromolecules under ATP hydrolysis, whereas the MFS transporters are single-polypeptide secondary carriers, capable of transporting small solutes in response to chemiosmotic ion gradients [28, 29]. Drug transporters of the ABC and MFS transporters in plant pathogenic fungi can function roles not only in the secretion of endogenous fungal pathogenic factors such as toxins but also in protecting against exogenous plant defense compounds such as phytoalexins, thereby playing an important role in virulence to plant [19, 30]. The *C. lunata* CX-3 genome encodes a great quantity of transporters (590) which is close to *C. lunata* m118 (594) and *B. maydis* C5 (593) (Additional file 1: Table S6). *C. lunata* CX-3 has 47 ABC transporters, close to 45 in *C. lunata* m118 and 46 in *B. maydis* C5. MFS transporters account for the highest proportion (252/590 in *C. lunata* CX-3, 251/594 in *C. lunata* m118 and 233/593 in *B. maydis* C5) (Additional file 1: Tables S1 and S6). 60-70% of MFS transporters (167/252 in CX-3, 171/251 in m118 and 158/233 in C5) and 80-85% of ABC transporters (40/47 in CX-3, 38/45 in m118 and 38/46 in C5) were identified as putative PHI genes in *C. lunata* CX-3, *C. lunata* m118, *B. maydis* C5 (Additional file 1: Table S2).

In the ABC transporters, the multidrug resistance (MDR) and the pleiotropic drug resistance (PDR) subfamilies function in resisting antifungal agents [31]. *C. lunata* CX-3 has 9 MDR subfamily of transporters, more than *C. lunata* m118 (6), *B. maydis* C5 (7) and the average of plant pathogens (8) (Additional file 1: Table S7). However, the number of PDR transporters in *C. lunata* CX-3 (10) is in line with *C. lunata* m118 (10), *B. maydis* C5 (10) and the average of plant pathogens (10). The two drug:H^+^ antiporter (DHA) subfamilies (DHA1 with 12 spanner and DHA2 with 14 spanner) of MFS transporter can make toxic compounds be secreted into outer environment [31]. DHA1 subfamily of transporters well exist in the *C. lunata* CX-3 genome (49 in CX-3 versus 52 in m118, 51 in C5 and an average of 39 in plant pathogenic fungi) (Additional file 1: Table S7). In comparison to DHA1, little DHA2 subfamily of transporters are present in *C. lunata* CX-3 (26) as well as *C. lunata* m118 (27) and *B. maydis* C5 (15), however which is higher than the average of plant pathogenic fungi. These results show that there are differences in gene family expansions occurred in DHA1, DHA2 and MDR among *C. lunata* CX-3, *C. lunata* m118 and *B. maydis* C5. Transcriptome sequencing of both the low virulent *C. lunata* strain and its virulence-increased variant showed that 16 transporters were up-regulated in the virulence-increased variant response to the selective pressure of host in the pathogen-plant interactions. Half of these transporters (8) were MFS transporters including CL01123, CL02605, CL03193, CL04682, CL04921, CL05805, CL08969 and CL09075, of which CL01123 and CL05805 were DHA1 subfamily of MFS transporters and CL04682 and CL04921 were DHA2 subfamily of MFS transporters. In the 16 transporters, there was only one MDR subfamily of ABC transporter (CL06556).

### Protein families for detoxification

Cytochrome P450 enzymes (CYPs) have a function with the conversion of hydrophobic intermediates of metabolisms and the detoxification of natural and environmental pollutants [32]. The genome sequencing of *C. lunata* facilitates the excavation of novel CYPs from *C. lunata*. Through the genome search against cytochrome P450 database [33], 137 P450 genes were identified in *C. lunata* CX-3, more than 106 in *C. lunata* m118, but less than 162 in *B. maydis* C5 (Additional file 1: Tables S1 and S8). 23 subfamilies of CYPs are presented in *C. lunata* CX-3 and/or *C. lunata* m118 but absent in *B. maydis* C5, and 13 subfamilies of CYPs are absent in both *C. lunata* CX-3 and *C. lunata* m118 but present in *B. maydis* C5, thus showing the significant differences in CYP families expansions in these genomes. Interestingly, there are a high percentage of PHI genes (about 70% or higher) in the CYPs of plant pathogenic fungi, for example, 112/137 (81%) in *C. lunata* CX-3, 95/107 (88%) in *C. lunata* m118 and 114/162 (70%) in *B. maydis* C5 (Additional file 1: Table S2), showing that most of CYPs are involved in the pathogen-plant interactions. CYP65 is the biggest subfamily of P450s both in *C. lunata* CX-3 (17), *C. lunata* m118 (12) and *B. maydis* C5 (21) (Additional file 1: Table S8). Additionally, CYP505 subfamily of P450s are present in these genomes (3 in CX-3, 2 in m118 and 4 in C5). It was reported that CYP65 catalyzed the epoxidation reaction in the mycotoxin trichothecene biosynthesis of *F. graminearum* [34], and CYP505 as well as CYP65 were involved in the fumonisin biosynthesis of a maize pathogen *F. verticillioides* [35, 36], suggesting that CYP505s and CYP65s in *C. lunata* and *B. maydis* probably be related to the toxin biosynthesis and thus play roles in fungal virulence.

### Virulence-associated signaling pathways

In fungal pathogenic lifestyle, G-protein-coupled receptors (GPCRs) are essential for plant recognition and pheromone/nutrient sensing [19]. They can transduce environmental signals by means of heterotrimeric G proteins into secondary messengers to regulate gene expression and ultimately cellular responses [37]. One GPCR of *M. grisea*, Pth11, mediates appressorium formation and virulence [38]. The *C. lunata* CX-3 genome contains a large number of GPCR-like genes with 129 in total and 51 putative Pth11-like GPCRs, which is similar to 139/54 in *C. lunata* m118 and but more than 102/30 in *B. maydis* C5 (Additional file 1: Tables S1 and S9). G-protein alpha subunit is a component of heterotrimeric G protein [39], and activates distinct downstream effectors and influence the pathogenicity [40, 41]. Notably, *C. lunata* CX-3 contains four G-protein alpha subunits (CL02298, CL05431, CL05929 and CL06671) that are PHI genes, and it was similarly found in *C. lunata* m118 and *B. maydis* C5 (Additional file 1: Table S2). The four G-protein alpha subunits showed 97% (CL02298), 92% (CL05431), 99% (CL05929) and 61% (CL06671) amino acid identities with heterotrimeric G protein alpha subunit subtype 2 of *S. turcica* (GenBank: AEJ38171), guanine nucleotide-binding protein alpha-3 subunit protein of *Neofusicoccum parvum* (GenBank: EOD45444), G-alpha subunit of *Phaeosphaeria nodorum* (GenBank: XP_001800368) and guanine nucleotide-binding protein alpha-3 subunit of *P. tritici-repentis* (GenBank: XP_001931840), respectively.

Likewise, three main signaling pathways (MAPK, cAMP and Ca), being mediated by protein kinases, control virulence-associated development of the fungal pathogen. Fungal protein kinases are classed into 9 groups, of which STE and CMGC (MAPK family) kinases are involved in MAPK pathway, AGC (PKA family) kinases in cAMP pathway, and CMGC (CLK and RCK family), CAMK and AGC (PKC family) kinases in Ca signaling pathway. The *C. lunata* CX-3 genome contains 153 protein kinases and it is slightly more than 147 in *C. lunata* m118 and 140 in *B. maydis* C5 and close to the average (154) of plant pathogenic fungi (Additional file 1: Tables S1 and S10). Because signal transduction plays a critical role in fungal development and infection [20], most of these protein kinases are highly homologous to gene-encoded proteins from PHI database (127/153 in CX-3, 124/147 in m118 and 114/140 in C5) (Additional file 1: Tables S1 and S2). Although there are no distinct differences in the number of protein kinases in the three genomes, the function of signal pathways regulated by protein kinases in different organisms are distinct. For example, Pmk1 MAPK pathway in *M. grisea* is involved in pathogenesis and regulates appressorium formation*,* however its homologous MAPK pathway in *S. cerevisiae* is related to both the pheromone signaling and filamentation.

*S. cerevisiae* has five MAPK pathways such as Fus3, Kss1, Hog1, Mpk1 and Smk1, which are involved in pheromone response, filamentous growth, osmoregulation, cell wall integrity and spore wall assembly, respectively [42]. *M. grisea* has three MAPK pathways such as Pmk1, Mps1 and Osm1, which are homologous to Fus3 or Kss1, Mpk1 and Hog1 pathways of *S. cerevisiae*, respectively [18]. Pmk1 pathway regulates appressorium formation, penetration and colonization; MPS1 pathway invasive growth, conidiation and penetration; OSM1 osmoregulation and stress response [18]. Blastp search analysis reveals that the homologues of MAPK pathways of *S. cerevisiae* and *M. grisea* were identified in the *C. lunata* CX-3 genome (Additional file 1: Table S11). The homologues of *pmk1* and *mps1*, *clk1* (CL06419) and *clm1* (CL11087) of *C. lunata*, were cloned and characterized in our previous study. *Clk1* gene influenced conidiospore formation, growth, cell degrading enzymes activity and virulence of *C. lunata* [9], which supported a result that homologues of *pmk1* were essential for fungal pathogenicity in all plant pathogens [43]. *Clm1* gene regulated cell-wall integrity, conidiospore formation, infection, cell degrading enzymes activity [10].

Cyclic AMP (cAMP) signaling pathway was involved in the induction of appressorium formation and turgor-driven process, therefore leading to plant infection [18]. cAMP can activate downstream effectors such as cAMP-dependent protein kinase (PKA) [44]. Catalytic subunit (CPKA) of PKA was required for appressorium maturation of *M. grisea* [45]. The *C. lunata* CX-3 genome contains two gene-encoded PKA catalytic subunits (CL03564 and CL01641), therefore proper studies of these genes will contribute to elaborating the cAMP signaling mediating appressorium formation.

Histidine kinase (HK) phosphorelay signaling as a major mechanism is used by some organisms (bacteria, slime molds, plants and fungi) to sense and adapt to their environment [20, 46]. In fungi, HK signaling mediates multi biological processes such as secondary metabolite biosynthesis, stress response, virulence and differentiation [46–48]. The pathway is two-component signaling pathway containing a sensor HK and a response regulator (RR). HKs well exist in *C. lunata* CX-3 (19), *C. lunata* m118 (19) and *B. maydis* C5 (20) and other plant pathogenic fungi (9 to 21) (Additional file 1: Table S10). It’s worth noting that all HKs are PHI genes.

### Virulence-associated secondary metabolite genes

One primary goal of studying the genome of a fungal pathogen is to identify secondary metabolites that are served as virulence factors such as host specific toxins (HST), non host specific toxin (NHST) and melanin [16]. The impact of HST/NHST on plant hosts was understood early, since they make the producing fungi be highly virulent to crops. *C. lunata* CX-3 is capable of producing diverse secondary metabolites such as NHST and melanin that aid in niche exploitation and pathogenicity. Although toxin and melanin are two key pathogenic factors in *C. lunata*, the core genes for their biosynthesis are not identified in *C. lunata* yet. The *C. lunata* CX-3 genome provides the feasibility to identify the core genes for melanin and toxin such as non-ribosomal peptide synthetase (NRPS) and polyketide synthase (PKS). It was reported previously that PKS1 and PKS2, two PKS of *B. maydis* C5, functioned as the biosynthesis of T-toxin, and HST1, one NPRS of *Bipolaris zeicola* played a key role in the HC-toxin biosynthesis. The number of core genes for secondary metabolites in *C. lunata* CX-3 (36) is close to the average of other plant pathogenic fungi (41) (Table 3). The *C. lunata* CX-3 genome encodes similar amounts of NRPS (6), PKS (16) and NRPS-PKS hybrid genes (HYBRID) (2) with *C. lunata* m118 (5 NRPS, 14 PKS and 2 HYBRID). While, the numbers of NRPS and PKS in the two *C. lunata* genomes are slightly less than *B. maydis* C5 (9 NRPS and 19 PKS), and the latter has no HYBRID. These results show that the differences in the number of NPRS and PKS between strains or species are consistent with their adapting to the diverse environments and hosts.

**Table 3:** **Numbers of backbone-genes for the biosynthesis of secondary metabolites in different pathogenic fungi**

Classifications	Fungal species									
	CX-3	m118	C5	CZM	PN	ST	PTR	MO	AF	FG
DMAT	1	2	3	1	2	2	0	3	8	0
HYBRID	2	2	0	1	1	2	1	5	2	0
NRPS	6	5	9	7	9	9	12	8	18	10
NRPS-Like	10	9	7	8	5	7	6	6	14	10
PKS	16	14	22	11	12	23	14	23	25	12
PKS-Like	1	1	3	2	9	3	6	2	3	2
Total	36	33	44	30	38	46	39	47	70	34

**Classifications:** DMAT, dimethylallyl tryptophan synthase; HYBRID, hybrid PKS-NRPS enzyme; NRPS, non-ribosomal peptide synthetase; PKS, polyketide synthetase;. **Fungal species:** CX-3, *C. lunata* CX-3; m118, *C. lunata* m118; C5, *B. maydis* C5; CZM, *C. zeae-maydis*; PN, *Phaeosphaeria nodorum*, ST, *S. turcica*; PTR, *P. tritici-repentis*, MO, *M. oryzae*; AF, *A. flavus*; FG, *F. graminearum. B. maydis* = *C. heterostrophus*.

**Figure 3:**
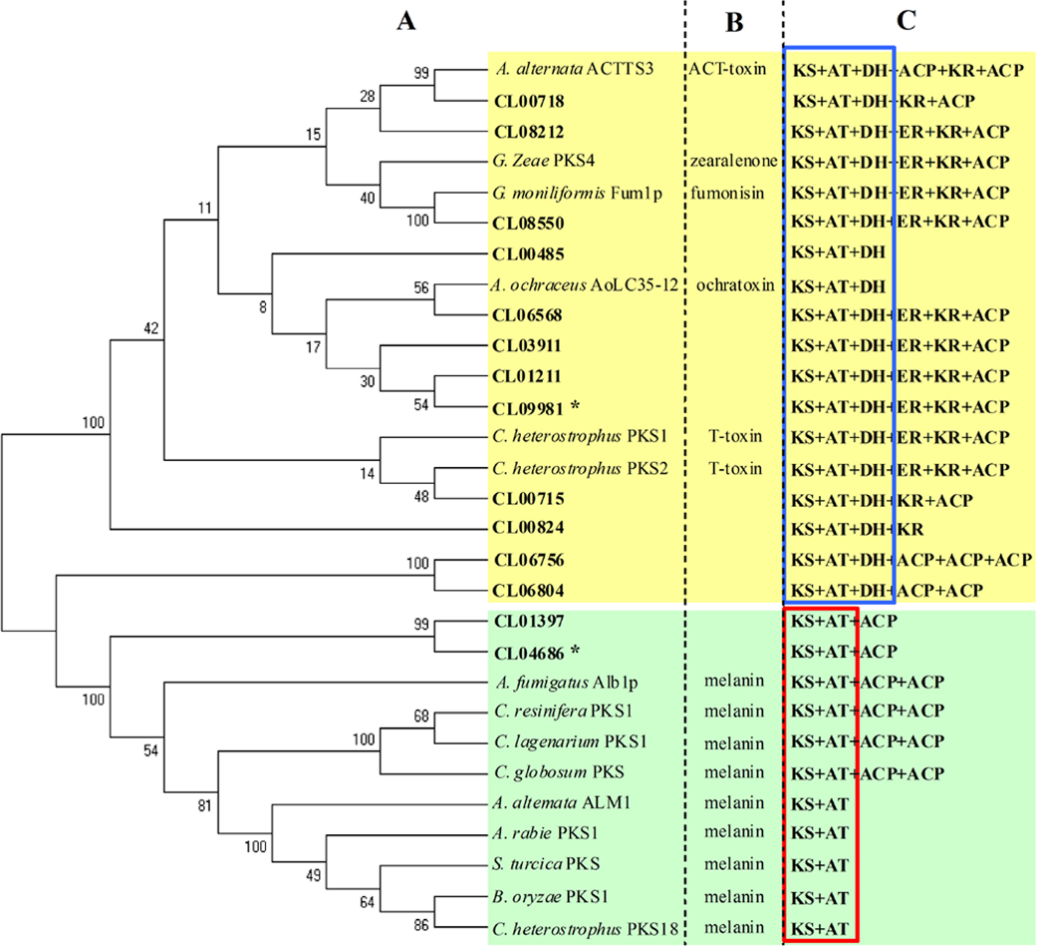
**Phylogenetic and domain analyses of** ***C. lunata*** **CX-3 and other fungal polyketide synthases (PKS). (A)** A neighbor-joining tree of ketoacyl CoA synthase (KS) domain sequences among fungi. **(B)** Toxin and melanin related to PKS in other fungi. **(C)** Domain analysis of the PKS. Domain definitions: KS, ketoacyl CoA synthase; AT, acyltransferase domain; DH, dehydratase domain; ER, enoyl reductase domain; KR, ketoreductase domain; ACP, acyl carrier protein domain; The accessions of other fungi PKSs are shown in the Materials and methods. *CL04686 and CL09981 are up-regulated in virulence-enhanced strain. *C. heterostrophus* = *B. maydis*.

*C. lunata* CX-3 PKSs were divided into different clusters based on phylogenetic analysis of the ketoacyl CoA synthase (KS) domain of PKSs with other known PKS for melanin or toxin biosynthesis as references (Figure 3A). There were differences in the domains of different PKSs in *C. lunata* CX-3 based on domain analysis, but the domains of *C. lunata* CX-3 PKSs were similar with known PKSs. Interestingly, PKSs were grouped into two kinds based on PKS domain components. One kind was reducing PKSs with KS, acyltransferase (AT) and dehydratase (DH) domains at least, including 12 *C. lunata* CX-3 PKSs and 6 other known PKSs being involved in different kinds of toxin biosynthesis such as *Alternaria alternata* ACT-toxin, *Gibberella zeae* zearalenone, *Gibberella moniliformis* fumonisin, *Aspergillus ochraceus* ochratoxin and *C. heterostrophus* T-toxin (Figure 3C). The other kind was non-reducing PKSs without DH domain, including 2 *C. lunata* CX-3 PKSs and 9 other known PKSs for melanin biosynthesis. It was suggested that *C. lunata* CX-3 has conserved PKSs related to the biosynthesis of toxin and melanin. Notably CL04686 and CL09981 both encoding PKSs were up-regulated in virulence-enhanced strain. However, phylogenetic and modular analyses suggested that the protein structures of *C. lunata* CX-3 NRPSs were obviously different from other known NRPSs being involved in the biosynthesis of mycotoxins such as HC-toxin of *Cochliobolus carbonum*, AM-toxin of *A. alternate*, gliotoxin of *Aspergillus fumigatus* and enniatin of *Fusarium equiseti* (Additional file 2: Figure S1).

### Small, cysteine-rich peptides and effector proteins

The small cysteine-rich proteins (SCRPs) were secreted directly into host plant cells and perform multiple biological functions such as host recognition or colonization [49, 50], the induction of host HR [51–53], pathogenicity [54], and antimicrobiosis [55, 56]. Some SCRPs as virulence effectors showed carbohydrate binding activity that not only facilitated fungal virulence by perturbing host cell signaling or interfering with host recognition of the pathogen or suppressing pathogen-associated molecular pattern (PAMP)-triggered immunity (PTI) [18, 57], but also induced effector-triggered immunity (ETI) governed by a gene-for-gene system in plants containing homologous resistance (*R*) proteins in the pathogen-host interaction [58]. A recently identified class of conserved effectors in fungi are LysM effectors that contain lysine motifs (LysMs) other than recognizable protein domains [59]. It is feasible to under-predict putative SCRPs in the *C. lunata* CX-3 genome due to the short length (<150 amino acid residues) of SCRPs, which contribute to extending the annotation of the *C. lunata* CX-3 genome with a specific search (see Materials and Methods). 76 potential SCRPs were found in the genome, ranging in size from 72 to 150 residues (Additional file 1: Table S12). Among these 76 predicted SCRPs in *C. lunata* CX-3, CL08250 (147 residues) with a LysM showed 34% of amino acid identity with Ecp6 protein of *Cladosporium fulvum*, which was a LysM-containing effector and virulence factor [60]. CL08356 (92 residues) containing an antifungal protein domain showed 48% of amino acid identity with an antifungal protein (GenBank: CAR79018) of *Fusarium avenaceum*. CL11021 (125 residues) containing a hydrophobin domain showed 45% of amino acid identity with a hydrophobin (GenBkan: ABY48863) from *B. maydis*. Several secreted hydrophobins as fungal effectors were examined for their roles in virulence such as *Hum3* and *Rsp1* of *U. maydis* [58, 61], fungal development and plant colonization such as *Mhp1* of *M. grisea* [62]. Additionally, although >150 residues in size of a secreted protein (CL01513, 234 residues), it contains a LysM (Additional file 1: Table S12) and shows 42% of amino acid identity with Ecp6 (GenBank: ACF19427) of *Passalora fulva*. As described above, cysteine-rich polypeptides such as CL01513, CL08250, CL08356 and CL11021 were served as potential candidates for pathogen effectors in *C. lunata* CX-3.

### Comparative transcriptome analysis for pathogenicity variation

In the previous works, under the successive selection pressure of resistant maize germplasm, *C. lunata* virulence was enhanced [4]. Although some hallmarks related to virulence variation were screened, the variation mechanism at the transcriptional level was not deeply understood yet. In order to further shed light on the molecular regulation mechanism for virulence differentiation of *C. lunata*, high-throughput RNA-Seq was performed to compare the transcriptional differences between *C. lunata* WS18 with low virulence and its virulence-enhanced variant WS18-Pob21-11 and to further screen the crux genes that involved in the virulence variation of *C. lunata* under the host selection pressures.

**Figure 4:**
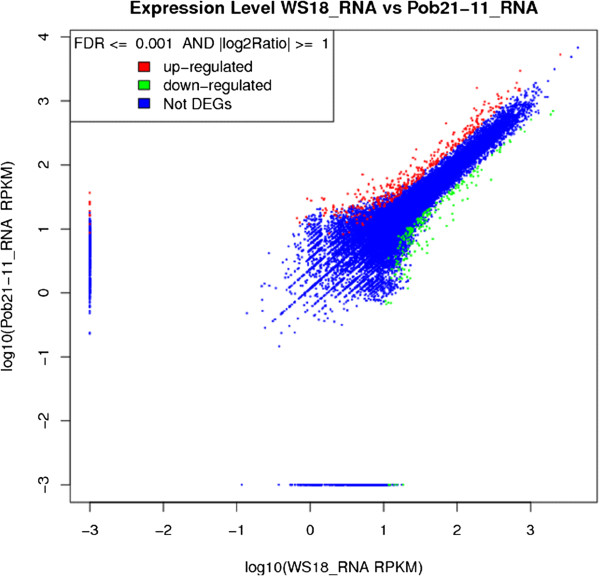
**Differentially expressed genes in the virulence-enhanced strain compared to the wild type.** The red and green color separately mean up-regulated and down-regulated unigenes, and the blue color means no differentially expressed unigenes. A total of 200 and 164 genes were significantly (PDR ≤ 0.001,) up-regulated and down-regulated in the virulence-enhanced strain WS18-Pob21-11.

**Figure 5:**
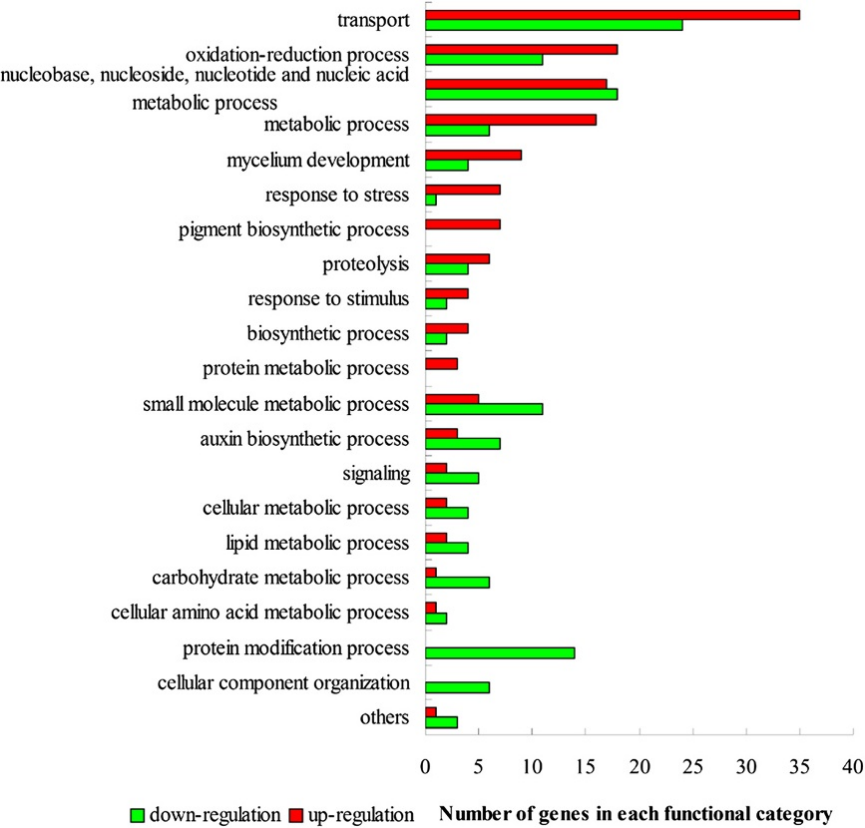
**Functional enrichment of differentially expressed genes in** ***C. lunata*** **WS18-Pob21-11 compared to WS18.**

>9.7 million tags were sequenced for each strain and 76.2% and 74.6% of predicted *C. lunata* CX-3 genes were expressed in WS18 and WS18-Pob21-11 respectively. However, the transcriptional profile of WS18-Pob21-11 was different from WS18 after successive host direction selection (Figure 4). A total of 200 genes including 32 putative PHI genes were significantly (PDR ≤ 0.001,) up-regulated and 164 genes including 35 putative PHI genes down-regulated in the virulence-enhanced strain (Additional file 1: Table S13), showing that *C. lunata* presented the obvious change at transcriptional regulation under the continued selective pressure from resistant host. In general, differential genes were involved in transport, oxidation-reduction process, metabolic process, mycelium development, response to stress, pigment biosynthetic process and protein metabolic process, etc., most of which were significantly up-regulated under the successive selection pressure from host. In contrast, almost all differentially expressed genes being involved in the carbohydrate metabolism, protein modification and cellular component organization were significantly down-regulated (Figure 5).

**Figure 6:**
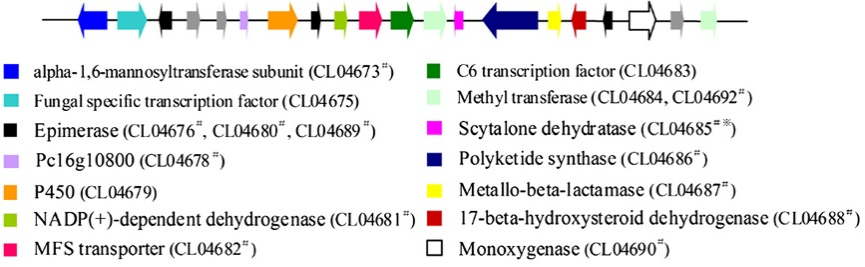
**Gene cluster for toxin synthesis in the** ***C. lunata*** **CX-3 genome.** ^※^Scytalone dehydratase involves in melanin synthesis. ^#^Genes were up-regulated in highly virulent *C. lunata* strain.

Like other plant pathogenic fungi, *C. lunata* could produce 1,8-dihydroxynaphthalene (DHN) melanin and toxin as two important pathogenic factors to make plant infected. In this study, it was found that the virulence-enhanced strain produced more melanin than the wild-type strain (data not shown), showing that the differential genes related to the biosynthesis of DHN-melanin were at lest partly responsible for the virulence variation under the selective pressures from host [4]. Responses to the selective pressure of host, CL04685 encoding scytalone dehydratase (SCD) were up-regulated PHI genes and it was involved in melanin synthesis [63, 64]. Therefore, it was proved that the pathway of melanin synthesis was related to virulence differentiation of *C. lunata*. Interestingly, it was found by Blastp of differential genes against the proteins sequences of the *C. lunata* CX-3 genome that 12 flanking genes of CL04685 (*scd*) in genome were up-regulated in WS18-Pob21-11, including CL04673 (alpha-1,6-mannosyltransferase subunit), CL04676 (epimerase), CL04678 (Pc16g10800), CL04680 (epimerase), CL04681 (NADP(+)-dependent dehydrogenase), CL04682 (MFS transporter), CL04686 (polyketide synthase), CL04687 (metallo-beta-lactamase), CL04688 (17-beta-hydroxysteroid dehydrogenase), CL04689 (epimerase), CL04690 (monoxygenase) and CL04692 (methyltransferase). Among the 12 flanking genes, the CL04682, CL04688 and CL04690 were PHI genes (Additional file 1: Table S13). Genes being involved in the biosynthesis of secondary metabolites were usually clustered [65], and the combination of CL04685 and its 12 flanking genes was similar to a sirodesmin biosynthesis-associated gene cluster of *Leptosphaeria maculans* [66] and a cercosporin-associated gene cluster of *Cercospora nicotianae* [67]. Thus, the 13 genes belonged to a gene cluster for the synthesis of secondary metabolites in *C. lunata* (Figure 6). In this cluster, CL04683 with no expression change has 27% of amino acid sequence identity with aflatoxin regulatory protein AflR (GenBank: AAM03003) which was involved in the regulation of aflatoxin clusters in *A. flavus* and *Aspergillus parasiticus* and sterigmatocystin cluster in *Aspergillus nidulans*, and 24% of amino acid sequence identity with cercosporin regulatory protein CTB8 (GenBank: DQ991510) of *C. nicotianae*. CL04682, a MFS transporter, has 61% of amino acid sequence identity with aflatoxin efflux pump (GenBank: XP_002844735) of *Arthroderma otae*. It was suggested that this cluster in *C. lunata* is a putative gene cluster for toxin biosynthesis of *C. lunata*. Therefore, the up-regulation of the cluster genes showed that they were involved in pathogenicity variation.

**Figure 7:**
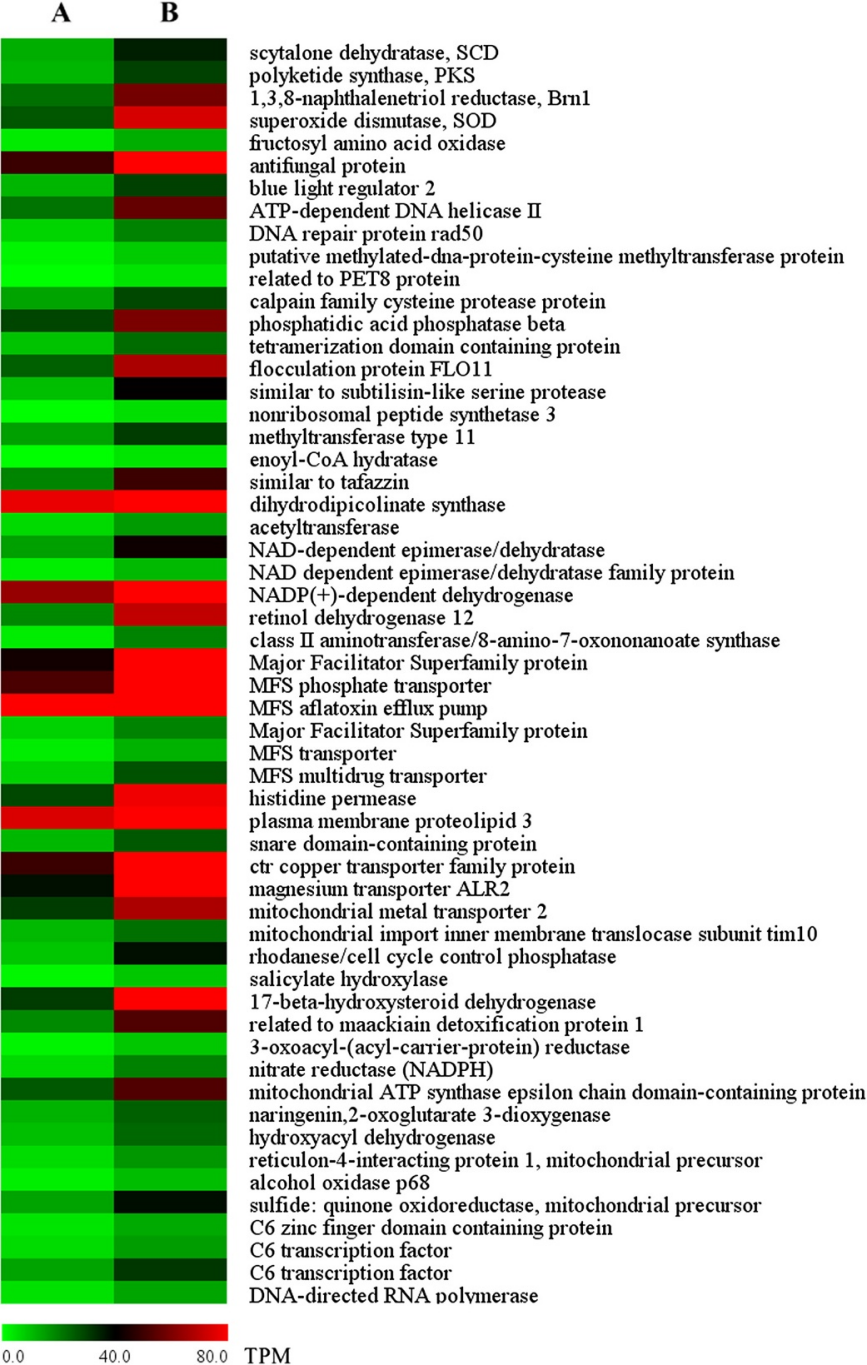
**Part of up-regulated genes related to the virulence variation in highly virulent** ***C. lunata*** **strain. A**: WS18, **B**: WS18-Pob21-11.

A complex network of regulatory and signaling components involved in regulation of morphogenesis and virulence [68, 69]. Therefore, although a gene cluster related to toxin and melanin biosynthesis played roles in the pathogenicity variation, other differently expressed genes were probably relative to the change, particularly up-regulated genes (Figure 7).

## Discussion

In this study, we reported the first genome sequence of *C. lunata*, an important pathogen of maize. Genome sequencing of the pathogen will make more contribution to the understanding of its evolutionary relationship with other pathogenic fungi, and efficiently screening of pathogenicity-associated genes as well as detection of its virulence variation under its interaction with host. It was showed that *C. lunata* CX-3 has significant differences from *C. lunata* m118 and *B. maydis* C5 in the number and sequence of genes-encoded proteins, although their evolutionary relationships are very close. As a plant pathogen, the *C. lunata* CX-3 genome like *B. maydis* contains about thousands of putative PHI genes (17.0% of all the genes) which are well known to be closely involved in the interaction with plants. Additionally, it was also found that the proportion of secretary proteins in *C. lunata* CX-3 was close to that in *C. lunata* m118 and *B. maydis* C5. Secreted proteins of pathogens have some crucial effectors responsible for mediating plant-pathogen interaction, some of which may play a role in the induction/suppression of plant resistance against pathogen infection [70, 71]. Transcriptional profile deriving from RNA-seq showed that the virulence variation of *C. lunata* was not only related to the up-regulation of genes probably being involved in the biosynthesis pathway of toxin and melanin, it also related to the up-regulation of other pathogenicity associated genes. In other words, multiple regulatory networks involved in the virulence differentiation of *C. lunata* response to the selective pressures from resistant hosts.

It was found by the phylogenomic analysis that the *Cochliobolus* lineage was diverged from other well known plant pathogenic fungi including *S. turcica*, *P. tritici-repentis* and *M. oryzae,* which was consistent with Leonard’s result [72]. Comparatively, *Cochliobolus* lineage was more similar to *S. turcica* and *P. tritici-repentis* than to *M. oryzae* in protein sequences. A great deal of expressed sequence tags (ESTs) sourced from the suppression subtractive hybridization (SSH) library of *C. lunata* revealed a similar trend that *C. lunata* has high sequence similarities with *S. turcica* and *P. tritici-repentis* [4], which would allow us more efficiently and more easily to find novel functional factors or mechanisms in *C. lunata* for attacking host plants. Furthermore the similar resistant genes could also be searched between mentioned pathogenic fungi and applied in a way to share homologous gene sources.

A wide range of plant pathogenic fungi exhibit a high degree of genetic variability through varied ways [4, 18], based on the PAMP models (pathogen–associated molecular pattern), in which a numerous effectors are involved in compatible or incompatible interaction between pathogen and host plants. In our case, small cysteine-rich proteins (SCRPs), for example, were speculated to be contributor to host immunity response if incompatible interaction of pathogen and maize resistant varieties happen, which could serve as effectors responsible for inducing host resistance response such as PTI and ETI [70].

Transposases encoded by transposons played a key role in the evolution of eukaryotic gene regulatory network, and coordinated and regulated eukaryotic gene expression [73]. Therefore serious pathogenic variation of *C. lunata* was attributed to the presence of a large amount of transposases in *C. lunata* [4]. The amount of transposases in *C. lunata* CX-3 is much larger than not only in *C. lunata* m118 and *B. maydis* C5, also in other Ascomycetes [20]. The phenomenon could be explained by high C to T and G to A transitions mediated by RIP in the *C. lunata* CX-3 genome during its sexual cycle [26], as also proved in many Ascomycetes [17, 19, 20, 74, 75]. Thus, it was speculated that shift of C:G to T:A might make the pathogen more frequently mutate in virulence differentiation of *C. lunata,* at least partly, suggesting that *C. lunata* CX-3 has a strong potential to evolve into other pathogenic types of strains.

*C. lunata* successively undergoes asexual and sexual stages in his life cycle like other fungi. So far, the asexual stage of *C. lunata* is well known, but its sexual stage is still poorly understood. In sexual stage, *C. lunata* probably survived in soil in the shape of ascospores [76], but this stage was infrequent in nature. The asexual stage of *C. lunata* is key in causing disease to host plant. During the interaction of pathogen-plant, *C. lunata* and other pathogenic fungi undergo several complex and crucial steps, including attachment to the plant surface, germination on the plant surface and formation of infection structures, penetration of the host and colonization of the host tissue, which are crucial to cause disease [77]. Pathogenic factors, including toxin, melanin, cell wall degrading enzymes (pectinase, cellulase, hemicellulase, protease, amylase and phospholipase), cutinase and hormone are essential for completing these processes. Furthermore, many gene-encoded proteins such as GPCRs, kinases, core genes for the biosynthesis of secondary metabolite, CYPs, ABC transporters and MFS transporters are directly or indirectly involved in these processes. Excitingly, a great many of these proteins had been identified in the *C. lunata* CX-3 genome by bioinformatics and most of these protein families were involved in the pathogen-host interaction (Additional file 1: Table S2), which facilitated the understanding of pathogenic mechanism of *C. lunata*. In the previous study, the chemical structure of non-host-specific toxin in *C. lunata* was identified successfully, and it was speculated based on the toxin structure that genes related to toxin biosynthesis may exist in genome with clustering status. It was found through the pathogen genome and transcriptome sequencing that alpha-1,6-mannosyltransferase, epimerase, P450, MFS transcripter, methyl transferase, polyketide synthase, monooxygenase and 17-beta-hydroxysteroid dehydrogenase etc. might be members of a gene cluster and responsible for the toxin synthesis, which was a very great progress on the identification of genes related to toxin biosynthesis, even though there was no idea that those genes how to work in synergistic way so far.

Although *C. lunata* has greatly close relation to *B. maydis* and both they are maize pathogens, there are distinct differences in pathogenicity-associated families. MFS transporters, G-protein coupled receptors, protein kinases and proteases families in *C. lunata* being involved in transport, signal transduction or degradation are expanded in relative to *B. maydis*. Cytochrome P450, lipases, glycoside hydrolases and polyketide synthases families for detoxification, hydrolysis or secondary metabolites biosynthesis are contracted compared to *B. maydis*, which are expected to be crucial for the fungal survival in varied stress environments.

Comparative transcriptional studies for the wild type strain with low virulence and its variant with high virulence provided a global realization of gene expression response to the directly selective pressures of resistant host plants, which would contribute to better understand the virulence differentiation of *C. lunata* and clarify the biosynthesis pathways of secondary metabolites such as toxin and melanin. Results suggested that toxin and melanin may share some biosynthesis related genes or regulation genes, and gene clusters for the toxin and melanin biosynthesis may overlap. Nevertheless toxin and melanin were synergistic pathogenic factors to maize, which might take place in virulence differentiation induced by host selective pressures. In other words, an unknown crossovers regulation mode of toxin and melanin may govern global virulence performance. For example, *brn1* (CL07173)-encoded 1,3,8-trihydroxynaphthalene reductase is not just involved in melanin biosynthesis but also mediates toxin biosynthesis in *C. lunata* [78].

Although the identification of PHI genes in the genome wide and pathogenicity variation analysis in transcriptome level were performed, there are still some limitations in this work. The identification of PHI genes and the family classification were based on Blastp search against corresponding databases. Nevertheless the function of homologous genes in different organisms may be different, thus the specific function of the interested genes especially PHI genes in *C. lunata* should be further confirmed experimentally.

## Conclusions

In conclusion, we report the genome sequence and comparative genome analysis, and conduct transcriptional regulation of pathogenicity variation in the plant pathogenic fungus *C. lunata*. The genome and transcriptome data should facilitate the identification of candidate genes and accelerate the molecular studies on biology, pathogenic mechanism and virulence differentiation of *C. lunata*. The candidate genes for plant-pathogen interaction are worthy of interest in *C. lunata*.

## Methods

### Fungal strains

*C. lunata* CX-3 strain was selected for genome sequence since it is highly virulent to maize and caused typical lesion on the maize leaves. Its colony morphology on PDA plate at 28°C is less aerial hyphae [9]. The strain is stored in our laboratory and used for the study of pathogenicity mechanism of the pathogen for ten years. The strain is maintained on potato dextrose agar (PDA) medium at 4°C or silicone beads at −20°C.

### Genome sequencing and assembly

The whole genome of *C. lunata* CX-3 strain was de novo sequenced using Illumina, the next generation sequencing technology. Two DNA libraries with 700 bp and 5 kb insert fragments were constructed and Paired-End sequenced with the Illumina Genome Analyzer at the Beijing Genomics Institute (BGI, Shenzhen, China). The genome sequence was assembled by SOAPdenovo software (BGI) [79].

### Gene prediction and annotation

The genes structures of the *C. lunata* CX-3 genome were predicted using Augustus software [80], with the annotated gene information of *M. grisea* as a reference. The predicted genes were inspected manually to maximize the accuracy of gene prediction. All questionable open reading frames were individually searched against the NCBI (reference proteins) refseq_protein database by Blastp [20]. Repetitive sequences were predicted using RepeatMasker 3.3.0 [81], and the database version and the search engine were 20110920 and rmblastn 2,2,27+, respectively. The potential secreted proteins were screened by combining Target 1.1 [82], SignalP 4.1 Server [83], TMHMM Server v. 2.0 [84] and Big-GPI softwares [85]. Small cystein-rich proteins (SCRPs) were identified from predicted scecreted proteins based on their expected sequence characteristics [86]. Secreted proteins with 20 to 150 amino acids and at least four cysteins were served as putative SCRPs.

### Orthology and phylogenomic analysis

Orthologous protein domains in fungi were used for phylogenomic analysis by the Inparanoid 7.0 database [87]. In total, 1328 orthologous proteins were screened out between used fungi with a cutoff *E* value of 1e-20 and aligned using Clustal W 2.1 [88]. The concatenated amino acid sequences was used to created a neighbor-joining tree using with TREE-PUZZLE program with the Dayhoff model [89]. The divergence time between species was estimated using r8s software with the Langley-Fitch medel [90] by calibrating with the origin of the Ascomycota at 500–650 million years ago [91].

### Protein family classifications

The protein families of *C. lunata* CX-3 genome were classified by Pfam analysis (http://pfam.sanger.ac.uk/). To identify virulence-associated genes, Blastp searches of the *C. lunata* CX-3 genome were performed against protein sequences in the pathogen-host interaction database (version 3.2, http://www.phi-base.org/) with a cutoff *E* value of 1e-5. G-protein-coupled receptors were screened through local Blastp against GPCRDB database (http://www.gpcr.org/7tm/) with best hits and further confirmed by searching seven transmembrane helices. Pth11-like GPCRs in *C. lunata* were identified by local Blastp searching against Pth11 with a cutoff *E* value of 1e-10 [74]. Carbohydrate-active enzymes were classified using local Blastp searching against the CAZy database (http://www.cazy.org/). Protease families and kinases were identified by local Blastp analysis respectively against the MEROPS peptidase database with a cutoff *E* value of 1e-20 and KinBase database with a cutoff *E* value of 1e-10 [92]. Transporters were analyzed through local Blastp against Transporter Classification Database with a cutoff *E* value of 1e-40 [93]. Cytochrome P450s were classified based on Blastp alignment against P450 database with a cutoff *E* value of 1e-10 (http://drnelson.uthsc.edu/CytochromeP450.html).

### Analysis of genes involved in secondary metabolism

PKS, NRPS and NRPS-PKS were identified through submitting the whole genome data to SMURF system [94]. Different PKS or NRPS proteins were submitted to SBSPKS database to modulate and extract conserved domains by Blast search [95]. Phylogenetic analysis for the conserved domains was conducted by aligning the domain sequences and creating the Neighbor-joined tree with MEGE 5.0 using a Dayhoff model with 1,000 of bootstrap method. PKSs in *C. lunata* CX-3 and *B. maydis* were used in this analysis. In addition, toxin-associated PKSs used in the analysis included *Gibberella zeae* PKS4 (GenBank: ABB90283) for the biosynthesis of zearalenones, *Aspergillus ochraceus* PKS (GenBank: AAT92023) for ochratoxin, *Gibberella moniliformis* Fum1p (GenBank: AAD43562) for fumonisin, *Alternaria alternate* ACTTS3 (GenBank: BAJ14522) for ACT-toxin, *B. maydis* PKS1 (GenBank: AAB08104) and PKS2 (GenBank: AAR90257) for T-toxin. The melanin-associated PKSs used in the analysis included *B. maydis* PKS18 (GenBank: AAR90272), *A. fumigatus* Alb1p (GenBank: ACJ13039), *Ceratocystis resinifera* PKS1 (GenBank: AAO60166), *Colletotrichum lagenarium* PKS1 (GenBank: BAA18956), *Chaetomium globosum* PKS (GenBank: AFP82905), *A. alternate* ALM1 (GenBank: BAK64048), *Ascochyta rabie* PKS1 (GenBank: ACS74449), *S. turcica* PKS (GenBank: AEE68981), *Bipolaris oryzae* PKS1 (GenBank: BAD22832). The mycotoxin-associated NRPSs used for phylogenetic and modular analyses included *Cochliobolus carbonum* NRPS (GenBank: AAA33023) for HC-toxin [96], *Fusarium equiseti* NRPS (GenBank: CAA79245) for enniatin [97], *Tolypocladium inflatum* NRPS (GenBank: CAA82227) for cyclosporin [98], *A. fumigatus* Glip (GenBank: AAW03307) for gliotoxin [99], and *A. alternate* NRPS (GenBank: AAF01762) for AM-toxin [100].

### Transcriptome analysis

*C. lunata* stains WS18 and its variant WS18-Pob21-11 were selected for transcriptome analysis using RNA-seq technology. Transcriptomes of WS18-Pob21-11 and WS18 were served as test group and control. *C. lunata* WS18 and its variant WS18-Pob21-11 obtained by the successive induction of resistant maize population Pob21 were lowly and highly virulent to Pob21, respectively [4]. To extract total RNA of WS18 and WS18-Pob21-11, their mycelia were cultured in potato-dextrose (PD) liquid medium with 140 rpm of shaking at 28°C for 96 h. The incubated medium was harvested by filtration with sterile cheese cloth, washed using sterilized Milli-Q and it was grinded in liquid nitrogen. Then, total RNA was extracted using Trizol™ Reagent (Invitrogen, USA) and treated with DNase I (Takara, Japan) for removing genome DNA with the recommended method by the manufacturer. Messenger RNA was extracted, cut off randomly and then reverse transcribed into cDNA.

A sequencing library was constructed by PCR amplification of cDNA for tag preparation. The tags were Paired-End sequenced using de novo sequencing technique with Illumina HiSeq™ 2000. Tags only containing adapter sequences were omitted, then the remaining tags were mapped to the *C. lunata* CX-3 genome or annotated genes [19]. For comparing expression of each mapped gene between samples, the abundance of each tag was transformed to the value of transcripts per million tags (TPM) [19]. The mapped genes with a cutoff of *P* ≤ 0.05 and FDR ≤0.001 were identified as significantly differential genes [101].

### Availability of supporting data

This Whole Genome Shotgun project has been deposited at DDBJ/EMBL/GenBank under the accession JFHG00000000 for *C. lunata* CX-3. The version described in this paper is version JFHG01000000. Phylogenetic data (alignments and phylogenetic trees) can be available from the Dryad Digital Repository: http://doi.org/10.5061/dryad.22v18.

## Electronic supplementary material

Additional file 1: **Comparative genomics analysis of** ***C. lunata.*** The file is composed by 13 tables in separate excel sheets, in which the additional information refer to comparative genomic properties and gene family analysis of *C. lunata* with other pathogenic fungi. **Table S1.** The number of genes for selected gene families in *C. lunata* and other Ascomycetes. **Table S2.** Major protein families involved in pathogen-host interaction in *C. lunata* and other Ascomycetes. **Table S3.** Putative transposase-encoding genes. **Table S4.** Glycoside hydrolase in *C. lunata* and other fungi. **Table S5.** Protease genes in different fungal genomes, classed by MEROPS families. **Table S6.** Transporters in *C. lunata* CX-3, *C. lunata* m118 and *B. maydis* C5. **Table S7.** Drug transporters of *C. lunata* and other fungi. **Table S8.** – Cytochrome P450 genes in different fungal genomes, classed by CYP families. **Table S9.** G-protein-coupled receptors in different fungal genomes. **Table S10.** The number of protein kinases in different fungal genomes. **Table S11.** Proteins for MAP Kinase pathway in the *C. lunata* CX-3 genome. **Table S12.** The pupative small, cysteine-rich peptides encoding genes in *C. lunata* CX-3 genome. **Table S13.** Differentially expressed genes in WS18-Pob21-11 compared to WS18*.* (XLS 146 KB)

Additional file 2: Figure S1: Phylogenetic and domain analyses of *C. lunata* CX-3 NRPSs compared with known NRPS from other fungi for mycotoxin biosynthesis. (DOC 2 MB)

## Abbreviations

MAPK: Mitogen activated protein kinase
PKS: Polyketide synthases
CX-3: *C. lunata* CX-3
m118: *C. lunata* m118
C5: *B. maydis* C5
GI: Genomic island
PAI: Pathogenicity island
ARI: Antibiotic resistance island
MYA: Million years ago
GH: Glycoside hydrolase
ABC: ATP-binding cassette
RIP: Repeat-induced point mutation
MFS: Major facilitator superfamily
MDR: Multidrug resistance
PDR: Pleiotropic drug resistance
DHA: Drug:H^+^ antiporter
CYP: Cytochrome P450 enzyme
GPCR: G-protein-coupled receptor
cAMP: Cyclic AMP
HK: Histidine kinase
HST: Host specific toxin
NHST: Non host specific toxin
NRPS: Non-ribosomal peptide synthetase
HYBRID: NRPS-PKS hybrid
SCRP: Small cysteine-rich protein
LysM: Lysin motif
PHI: Pathogen-host interaction
PAMP: Pathogen associated molecular pattern
PTI: PAMP-triggered immunity
ETI: Effector-triggered immunity
TPM: Transcripts per million tags.
