# Supplementary material for: Genome sequence and virulence variation-related transcriptome profiles of Curvularia lunata, an important maize pathogenic fungus
Source: BMC Genomics. 2014 Jul 24;15(1):627. doi: 10.1186/1471-2164-15-627 (PMC4124159; doi:10.1186/1471-2164-15-627)
Supplement: Supplementary file 2 — Additional file 2: Figure S1: Phylogenetic and domain analyses of C. lunata CX-3 NRPSs compared with known NRPS from other fungi for mycotoxin biosynthesis. (DOC 2 MB) [file 12864_2014_6326_MOESM2_ESM.doc]

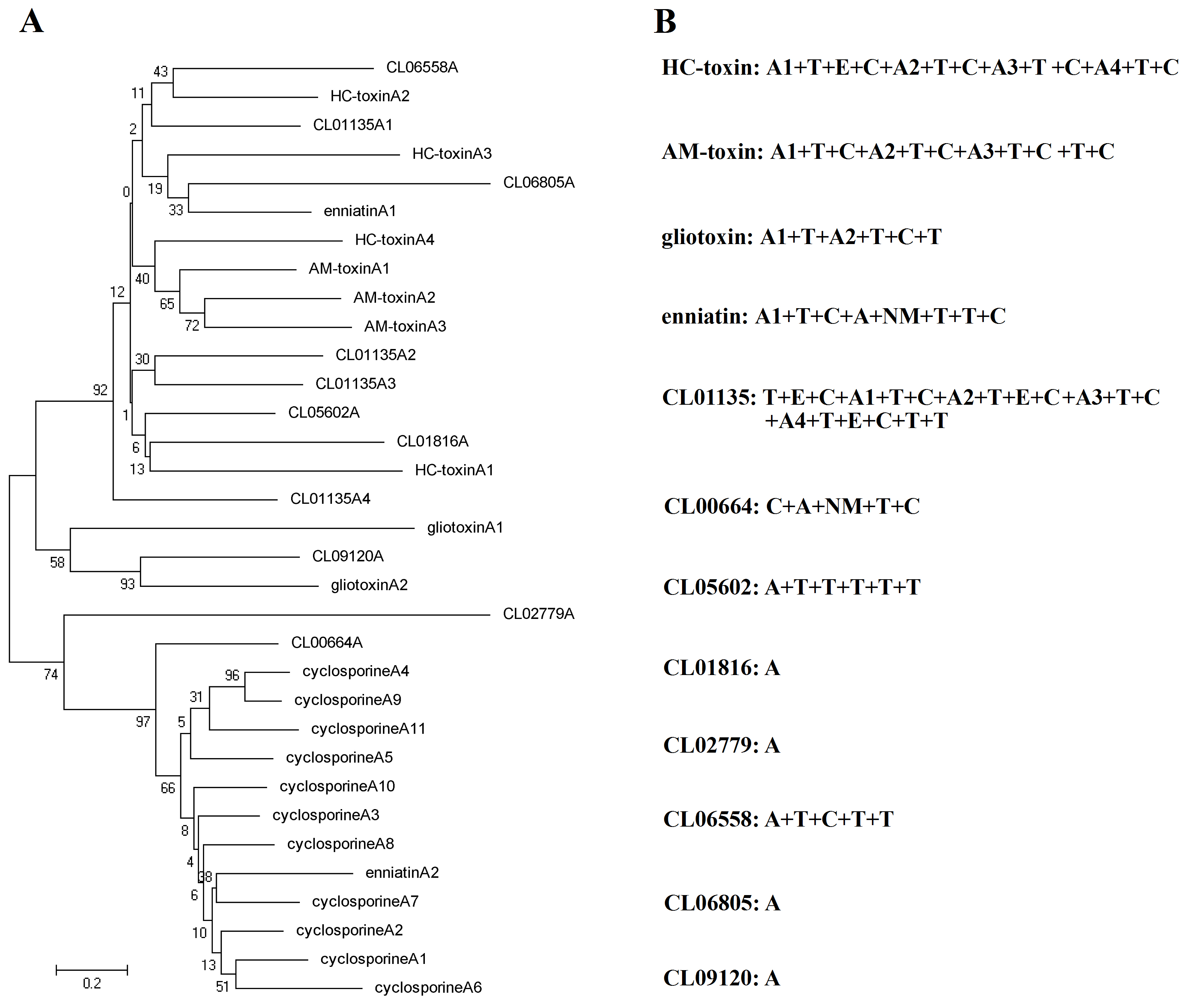


**Figure S1. Phylogenetic and domain analyses of *C. lunata* CX-3 NRPSs compared with known NRPSs from other fungi for mycotoxin biosynthesis.** (A) A neighbor-joining tree of adenylation (A) domain sequences of NRPSs. (B) Domain analyses of *C. lunata* CX-3 NRPSs with known mycotoxin-related NRPSs. NRPSs for known mycotoxin in the analysis include *C. carbonum* NRPS (GenBank: AAA33023) for HC-toxin, *F. equiseti* NRPS (GenBank: CAA79245) for enniatin, *T. inflatum* NRPS (GenBank: CAA82227) for cyclosporin, *A. fumigatus* Glip (GenBank: AAW03307) for gliotoxin and *A. alternate* NRPS (GenBank: AAF01762) for AM-toxin. Domain definitions: A, adenylation; T, thiolation; E, epimerization; C, condensation; NM, N-methylation.
